# Supplementary material for: Deficiency in intestinal epithelial O‐GlcNAcylation predisposes to gut inflammation
Source: EMBO Mol Med. 2018 Jun 25;10(8):e8736. doi: 10.15252/emmm.201708736 (PMC6079539; doi:10.15252/emmm.201708736)
Supplement: Supplementary file 1 — Appendix [file EMMM-10-e8736-s001.pdf]

Appendix

Deficiency in Intestinal Epithelial O-GlcNAcylation Predisposes to Gut Inflammation

Ming Zhao[1,2,10], Xiwen Xiong[1,10], Kaiqun Ren[2,3], Bing Xu[4], Meng Cheng[2], Chinmayi Sahu[2], Kaichun Wu[4], Yongzhan Nie[4], Zan Huang[2,5,6], Richard S. Blumberg[7], Xiaonan Han[8,9], Hai-Bin Ruan[1,2,*]

[1] School of Forensic Medicine, Xinxiang Medical University, Xinxiang, Henan, China

[2] Department of Integrative Biology and Physiology, University of Minnesota Medical School, Minneapolis, Minnesota 55455, USA[3] College of Medicine, Hunan Normal University, Changsha, Hunan, China

[4] State Key Laboratory of Cancer Biology & Institute of Digestive Diseases, Xijing Hospital, The Fourth Military Medical University, Xi'an, Shaanxi, China

[5] Laboratory of Gastrointestinal Microbiology, Jiangsu Key Laboratory of Gastrointestinal Nutrition and Animal Health, College of Animal Science and Technology, Nanjing Agriculture University, Nanjing, Jiangsu 210095, China

[6] National Center for International Research on Animal Gut Nutrition, Nanjing Agriculture University, Nanjing, Jiangsu 210095, China

[7] Division of Gastroenterology, Department of Medicine, Brigham and Women's Hospital, Harvard Medical School, Boston, Massachusetts 02115, USA

[8] Division of Gastroenterology, Hepatology, and Nutrition, Cincinnati Children's Hospital Medical Center, Cincinnati, Ohio, USA

[9] Key Laboratory of Human Disease Comparative Medicine, the Ministry of Health; Institute of Laboratory Animal Science, Chinese Academy of Medical Science (CAMS) and Peking Union Medical College (PUMC), Beijing, China

[10] These authors contributed equally to this study.

[*] Correspondence: Hai-Bin Ruan, PhD, 2261 6th St SE, Minneapolis, MN 55455, USA. Email: hruan@umn.edu. Tel: 612-301-7686; Fax: 612-301-1229.

Table of Content

Appendix Table S1 3

Appendix Table S2 4

Appendix Table S1

Specimen #	Pathological Status	Clinical Diagnosis	Tissue Site	Patient Blind ID	Patient Age	Gender
T140980_1	Non-Malignant, Diseased	Ulcerative colitis	Colon	15996	47	Male
T130806_6	Non-Malignant, Diseased	Ulcerative colitis	Colon	14729	52	Female
T130647_1	Non-Malignant, Diseased	Ulcerative colitis	Colon	14963	19	Female
T130391_1	Non-Malignant, Diseased	Ulcerative colitis	Colon	14831	46	Male
T022769_4	Non-Malignant, Diseased	Crohn's disease	Colon	6077	49	Male
T040385_4	Non-Malignant, Diseased	Crohn's disease	Colon	7085	44	Male
T041358_3	Non-Malignant, Diseased	Crohn's disease	Colon	7565	12	Male
T050520_5	Non-Malignant, Diseased	Crohn's disease	Colon	7916	56	Male
T121438_2	Non-Malignant	Normal adjacent tumor	Colon	14142	50	Female
T121423_3	Non-Malignant	Normal adjacent tumor	Colon	14077	64	Female
T121282_3	Non-Malignant	Normal adjacent tumor	Colon	14376	70	Male
T121135_2	Non-Malignant	Normal adjacent tumor	Colon	13945	71	Female

Appendix Table S2

Specimen #	Clinical Diagnosis	Tissue Site	Patient Age	OGT score	RL2 score	Geboes score	GHA score
T041173_4	Ulcerative colitis (disorder)	Colon	60	2.7	3.1	15	
T050561_3	Ulcerative colitis (disorder)	Rectum	18	1.3	N.D	15	
T050088_3	Ulcerative colitis (disorder)	Colon	25	2.0	1.1	17	
T050423_4	Ulcerative colitis (disorder)	Sigmoid colon	42	2.2	2.3	15	
T060894_4	Ulcerative colitis (disorder)	Colon	71	1.9	2.1	16	
T061024_3	Ulcerative colitis (disorder)	Rectum	65	2.3	1.0	13	
T060893_4	Ulcerative colitis (disorder)	Colon	26	2.8	2.1	9	
T122874_9	Ulcerative colitis (disorder)	Colon	52	1.3	2.5	16	
T130418_5	Ulcerative colitis (disorder)	Colon	40	2.9	2.8	16	
T141016_1	Ulcerative colitis (disorder)	Rectum, NOS	21	N.D	N.D	17	
T000531	Crohn's disease (disorder)	Small bowel	36	1.3	N.D		11
T010365	Crohn's disease (disorder)	Colon	37	N.D	2.0		15
T031083_2	Crohn's disease (disorder)	Ileum	32	1.0	2.2		10
T041484_4	Crohn's disease (disorder)	Colon	28	1.5	2.4		16
T051139_4	Crohn's disease (disorder)	Colon	33	2.5	2.3		2
T071143_3	Crohn's disease (disorder)	Colon	34	N.D	2.4		12
T131043_7	Crohn's disease (disorder)	Colon	33	N.D	N.D		3
T150465_1	Crohn's disease (disorder)	Colon	56	N.D	N.D		3
T180055_2	Crohn's disease (disorder)	Colon	38	2.6	1.8		7
T150002_1	Normal adjacent to Adenocarcionoma	Colon	58	2.6	4.0	1	1
T150319_6	Normal adjacent to Adenocarcionoma	Colon	75	2.2	3.7	2	2
T140180_1	Normal adjacent to Adenocarcionoma	Colon	50	2.9	3.3	1	1
T140130_3	Normal adjacent to Adenocarcionoma	Colon	46	4.0	3.6	1	3

T150374_3	Normal adjacent to Adenocarcionoma	Colon	44	3.0	2.6	2	0
T150429_3	Normal adjacent to Adenocarcionoma	Colon	74	3.2	2.9	4	4
T150591_3	Normal adjacent to Adenocarcionoma	Colon	44	2.0	3.0	0	0
T150690_1	Normal adjacent to Adenocarcionoma	Colon	39	4.0	3.2	2	3
T151125_1	Normal adjacent to Adenocarcionoma	Colon	58	3.8	3.8	0	1
T140390_1	Normal adjacent to Adenocarcionoma	Colon	69	2.0	2.9	1	0
