# Supplementary material for: Deficiency in intestinal epithelial O‐GlcNAcylation predisposes to gut inflammation
Source: EMBO Mol Med. 2018 Jun 25;10(8):e8736. doi: 10.15252/emmm.201708736 (PMC6079539; doi:10.15252/emmm.201708736)
Supplement: Supplementary file 2 — Expanded View Figures PDF [file EMMM-10-e8736-s002.pdf]

Expanded View Figures

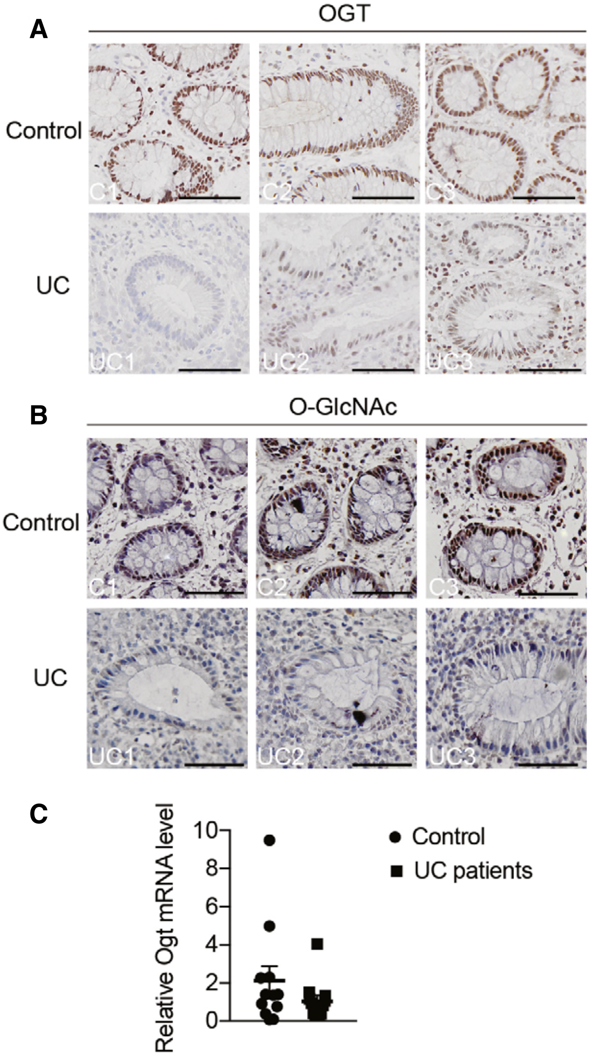

Figure EV1. Defective O-GlcNAc signaling in epithelial cells of Chinese UC patients.

A, B Representative images of OGT (A) and O-GlcNAc (B) immunohistochemistry in colon tissues from Chinese normal controls and UC subjects. Scale bars = 50 µm.

C mRNA levels of *OGT* in the colon from Chinese healthy and UC subjects (n = 12).

Data information: Data are represented as scatter dot plots with lines at mean and SEM.

© 2018 The Authors

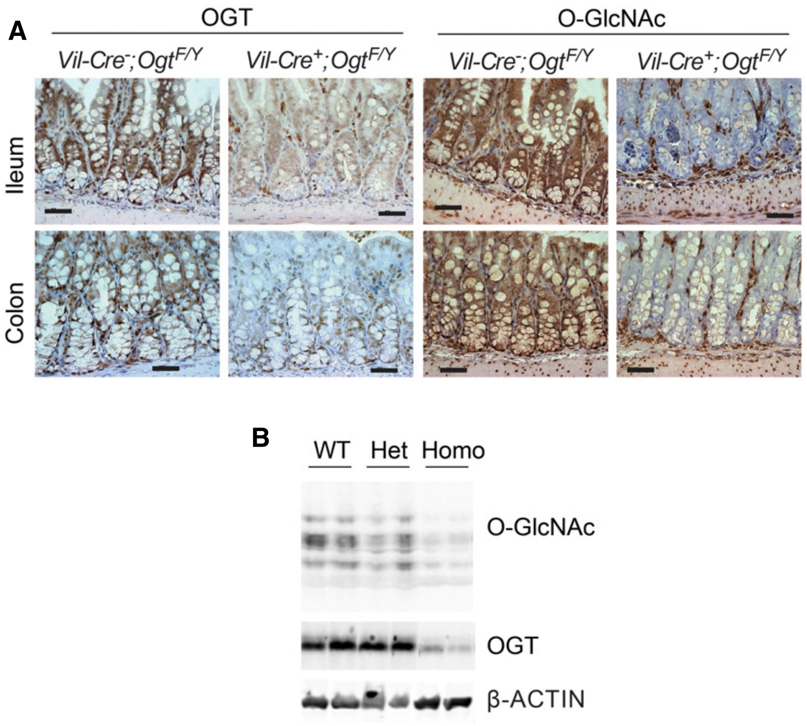

Figure EV2. Knockout specificity and efficiency in *Vil-OGT* KO mice.

A Immunostaining of OGT and O-GlcNAc in the ileum and colon sections from male wild-type and *Vil-Ogt* KO mice. Scale bars = 50 μm.

B Immunoblotting of O-GlcNAc and OGT in the colon of female wild-type and *Vil-Ogt* KO mice.

Source data are available online for this figure.

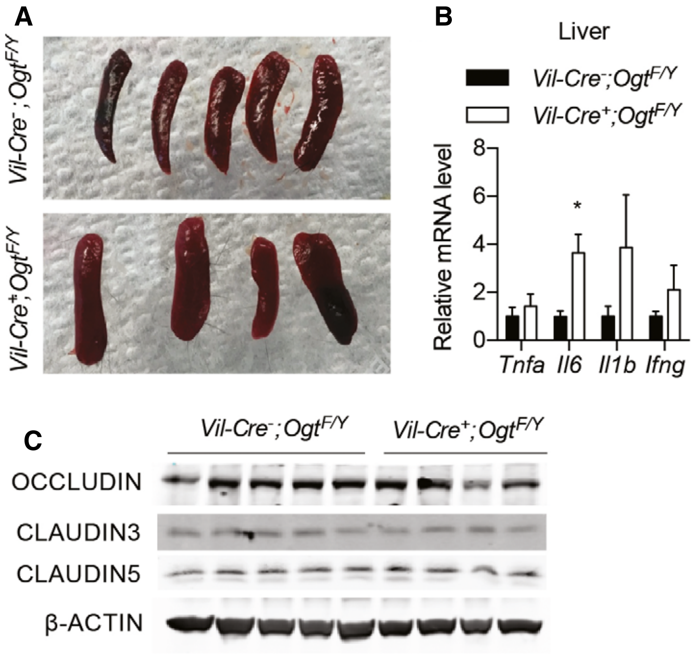

Figure EV3. Systemic inflammation in *Vil-Ogt* KO mice.

A, B Gross morphology of spleen (A) and RT–qPCR of inflammatory markers in liver (B) from male wild-type and *Vil-Ogt* KO mice (WT n = 4, KO n = 6, *Il6* P = 0.0271).

C Immunoblotting of protein markers of tight junction including OCCLUDIN, CLAUDIN 3 and 5 in colon. The blots were from the same experiment as shown in Fig 7A.

Data information: Data are represented as mean ± SEM. $^*P < 0.05$ by two-tailed t-test.

Source data are available online for this figure.

 © 2018 The Authors

Figure EV4. Changes in microbial composition in *Vil-Ogt* KO mice.

A Phylogenetic diversity of fecal bacteria from mice that were individually housed at UMN (WT n = 5, KO n = 4) or Yale (n = 5).

B Differentially abundant taxomonic clades analyzed by LEfSe.

C, D Abundance histogram plots of taxonomic groups detected by LEfSe that were downregulated (C) or upregulated (D) in *Vil-Ogt* KO mice (n = 4–5).

E–H Antibiotic-treated mice were transplanted with gut microbiota from control or *Vil-Ogt* KO mice (n = 5). (E) PCoA plot of unweighted UniFrac distance of bacterial communities. (F) H&E staining of colon. Scale bars = 100 μm. (G) Levels of serum FITC-dextran. (H) Albumin from fecal samples.

Data information: Data are represented as mean ± SEM.

© 2018 The Authors

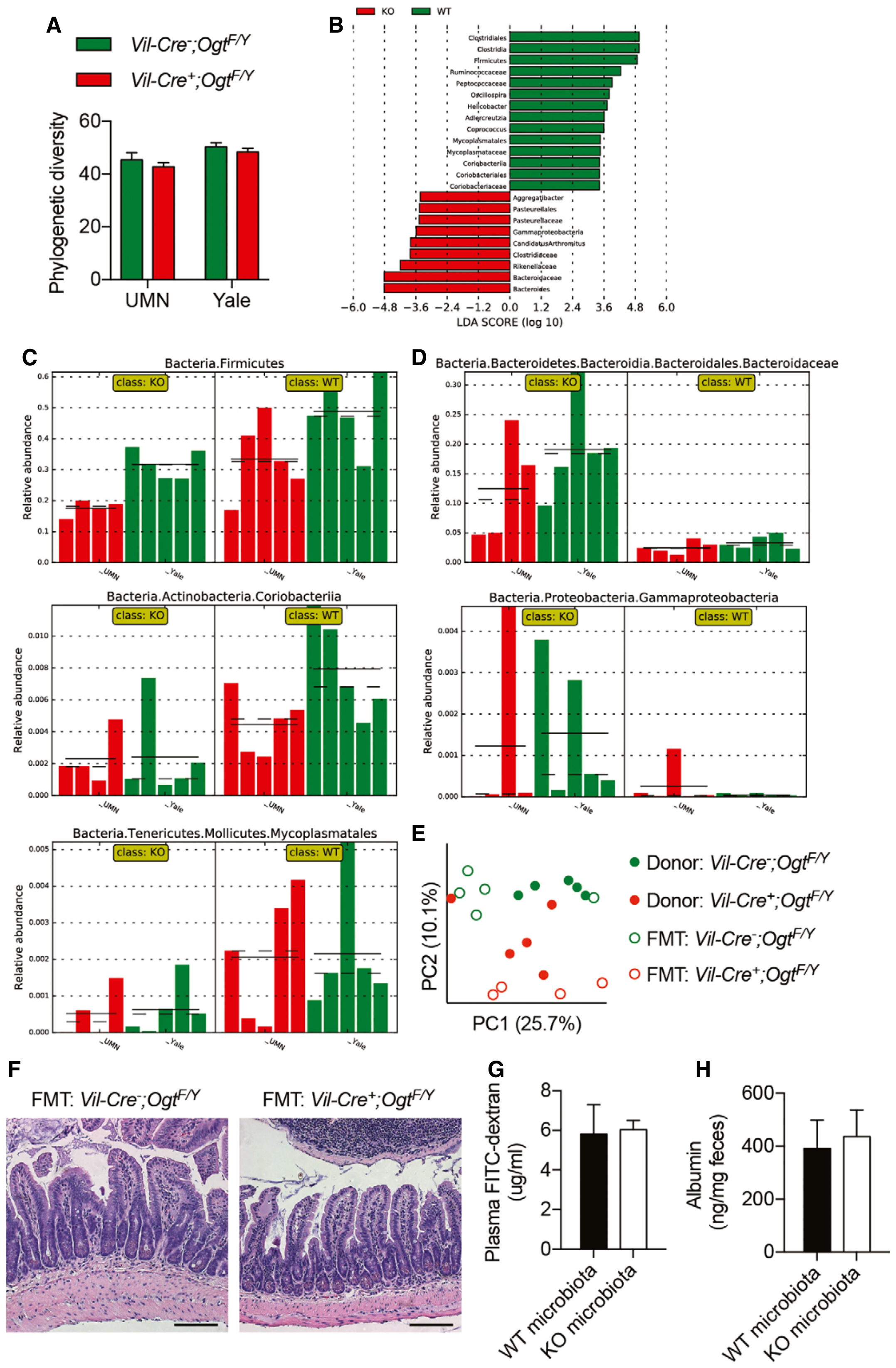

Figure EV4.

 © 2018 The Authors

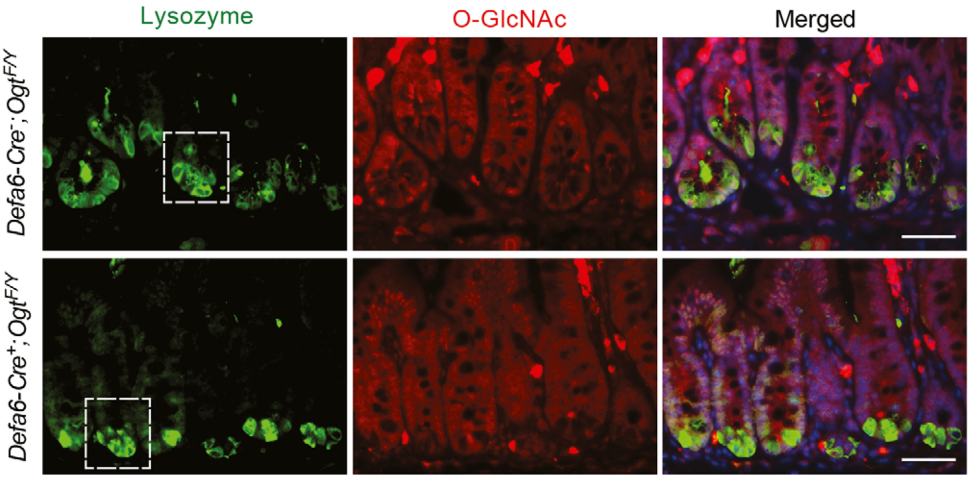

Figure EV5. Specific knockout of OGT in Paneth cells.

Immunostaining of protein O-GlcNAcylation in Paneth cells (marked by lysozyme staining) from wild-type and *Defa6-Ogt* KO mice, showing the knockout specificity/efficiency of OGT and the reduction of Paneth cell numbers. Zoom-in view of the regions indicated by the rectangles was shown in Fig 6E. Scale bars = 50 µm.

© 2018 The Authors
