# Supplementary figures and images for: Deficiency in intestinal epithelial O‐GlcNAcylation predisposes to gut inflammation
Source: EMBO Mol Med. 2018 Jun 25;10(8):e8736. doi: 10.15252/emmm.201708736 (PMC6079539; doi:10.15252/emmm.201708736)

Fig EV2

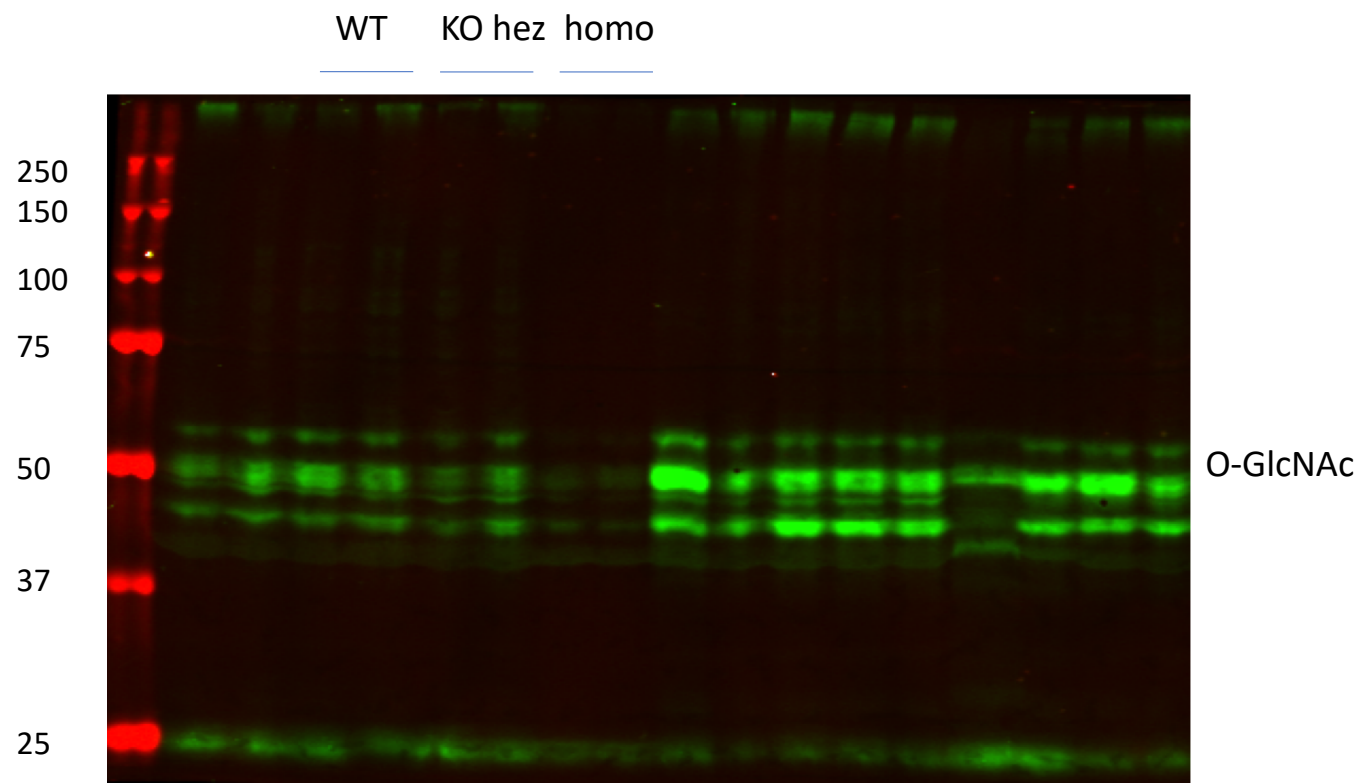

Fig EV2

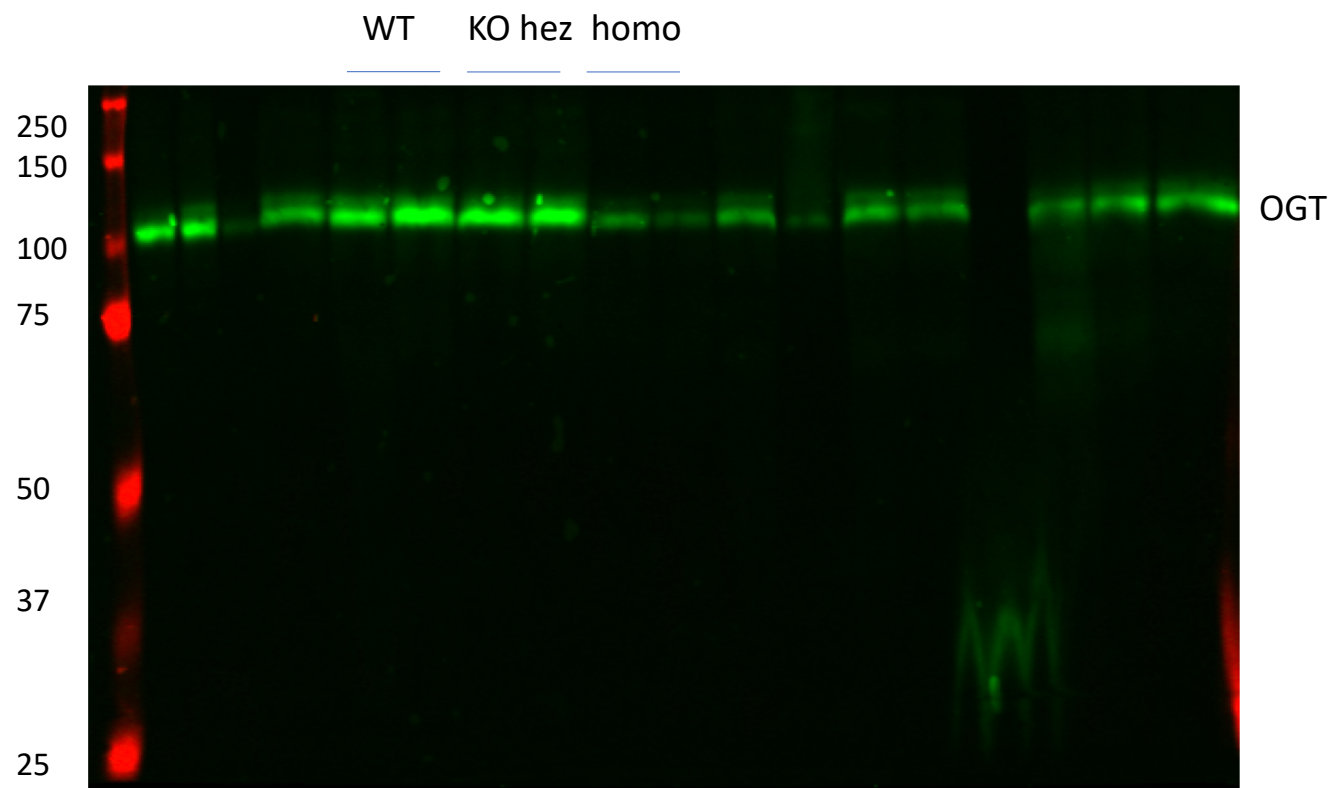

Fig EV2

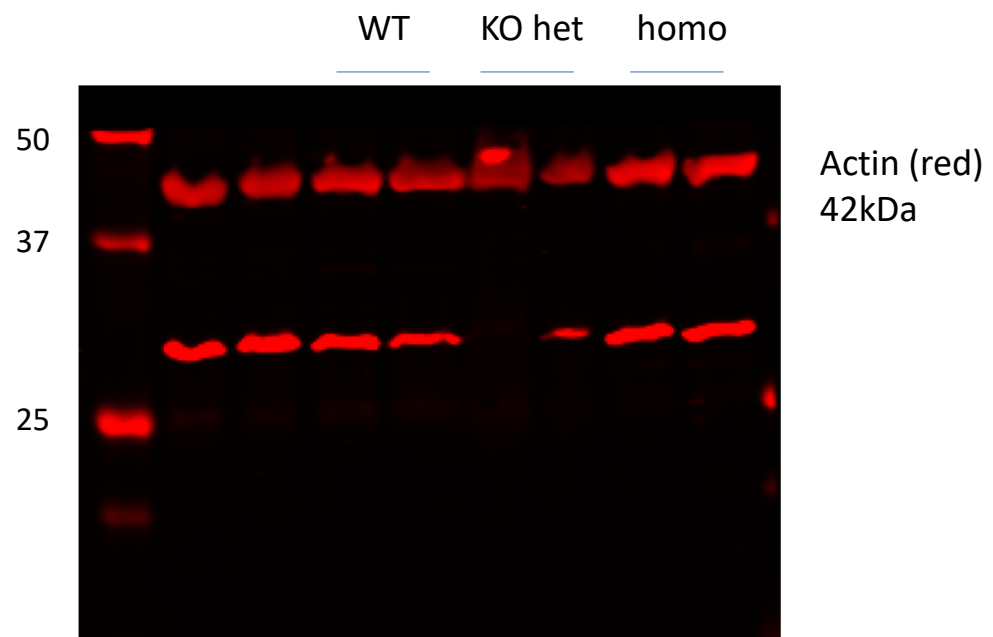

Supplement: Supplementary file 4 — Source Data for Expanded View [file EMMM-10-e8736-s007.zip › EV_source_data/Source_data_of_Fig_EV2B.pdf]

Fig EV3

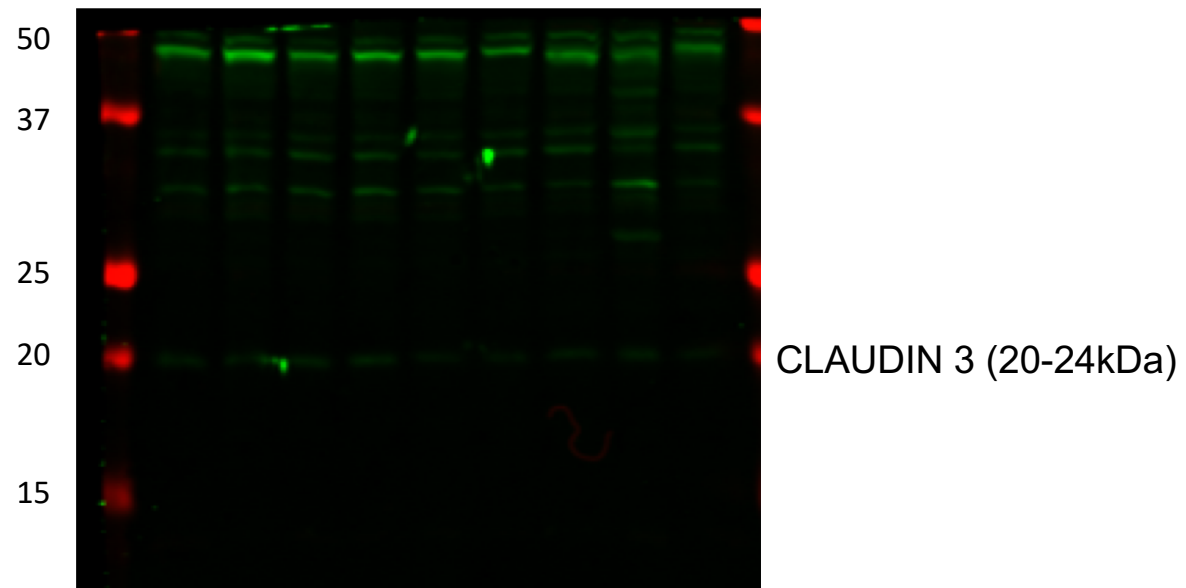

Fig EV3

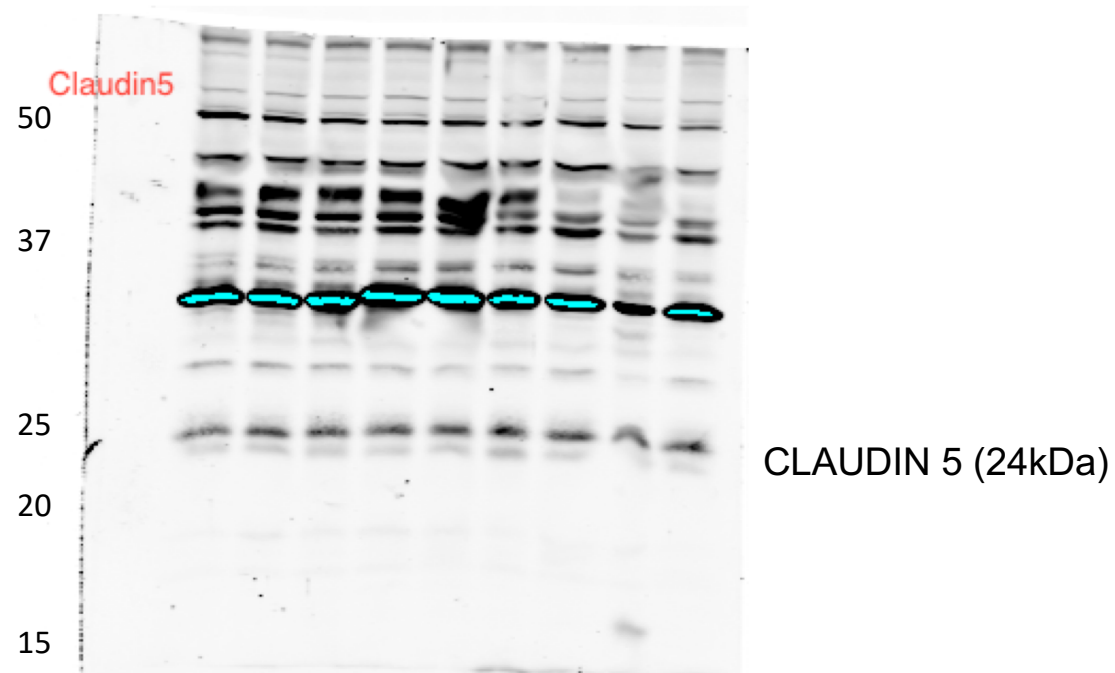

Fig EV3

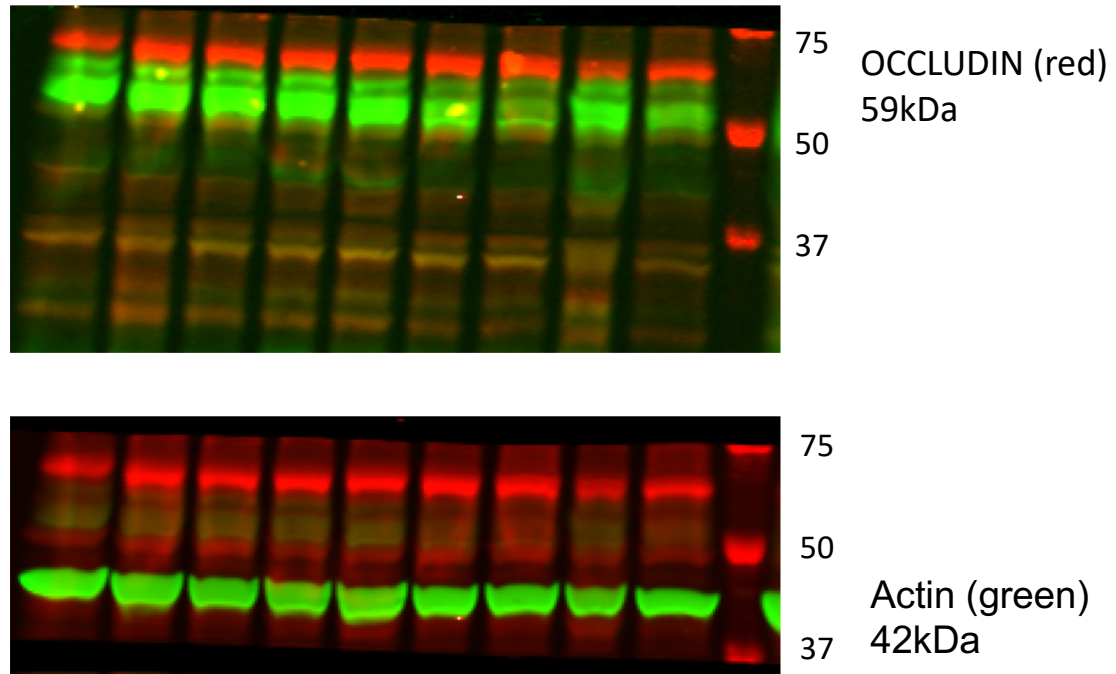

Supplement: Supplementary file 4 — Source Data for Expanded View [file EMMM-10-e8736-s007.zip › EV_source_data/Source_data_of_Fig_EV3C.pdf]

Fig 4D

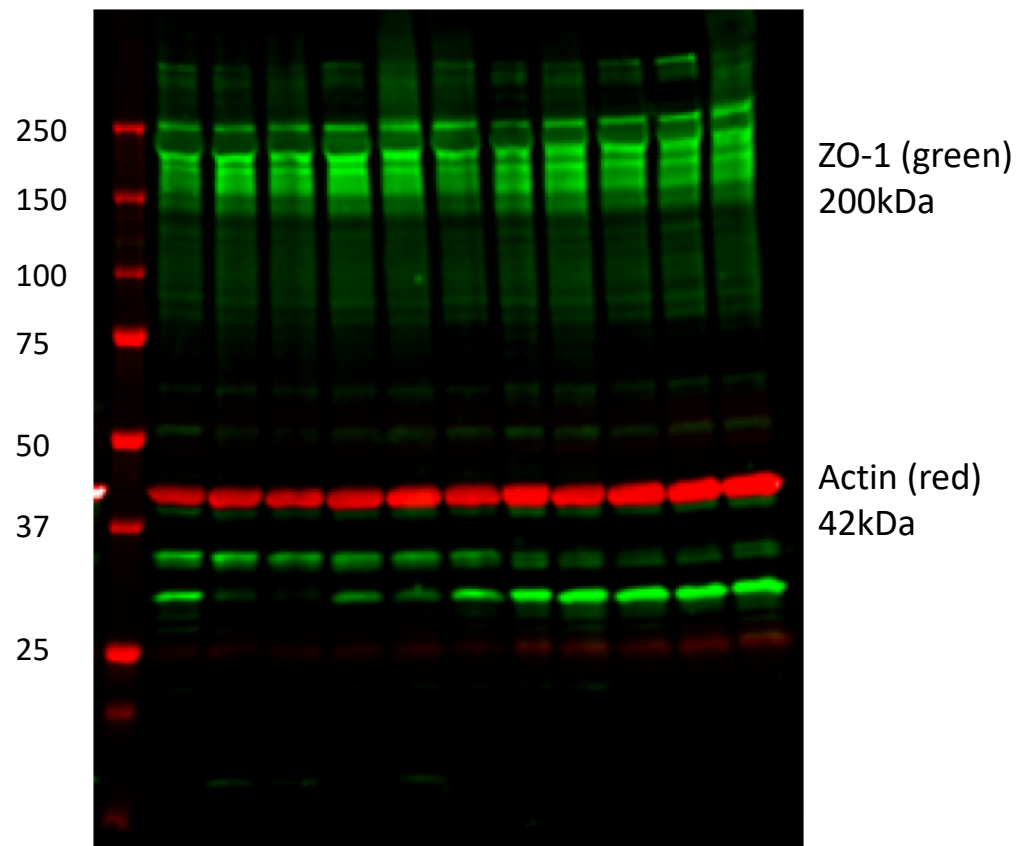

Fig 4D

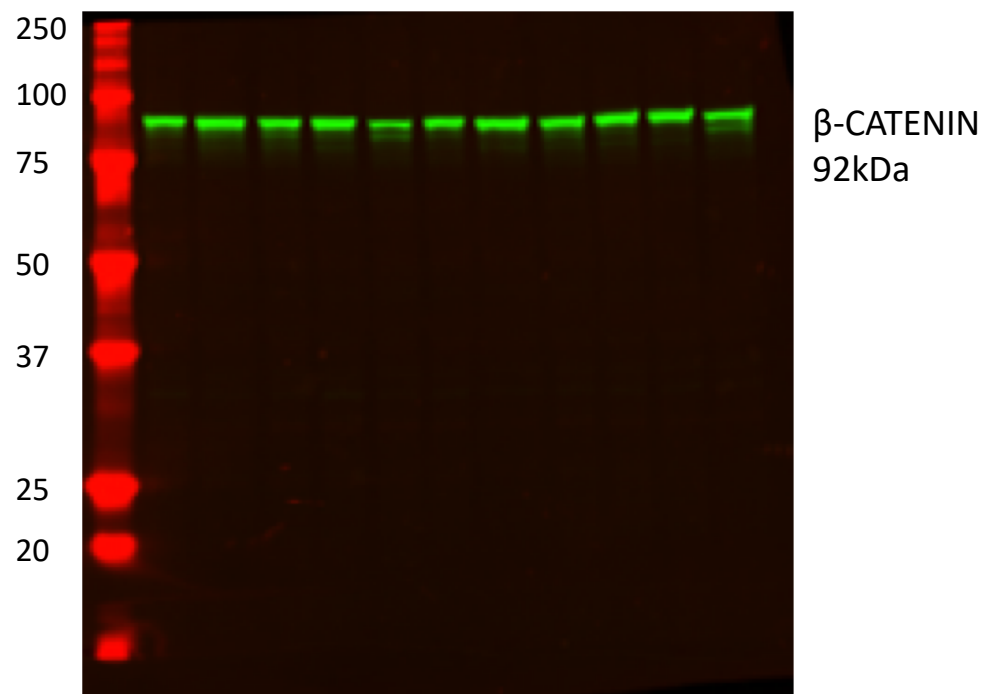

Fig 4D

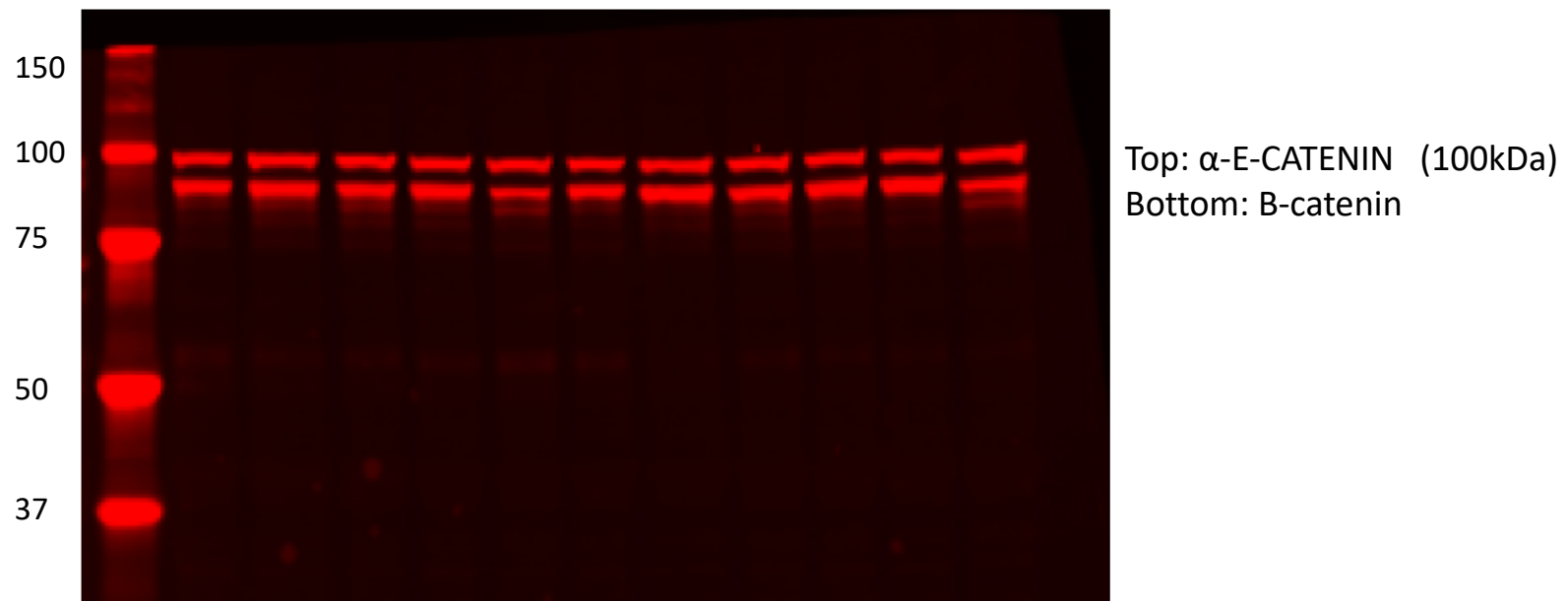

Fig 4D

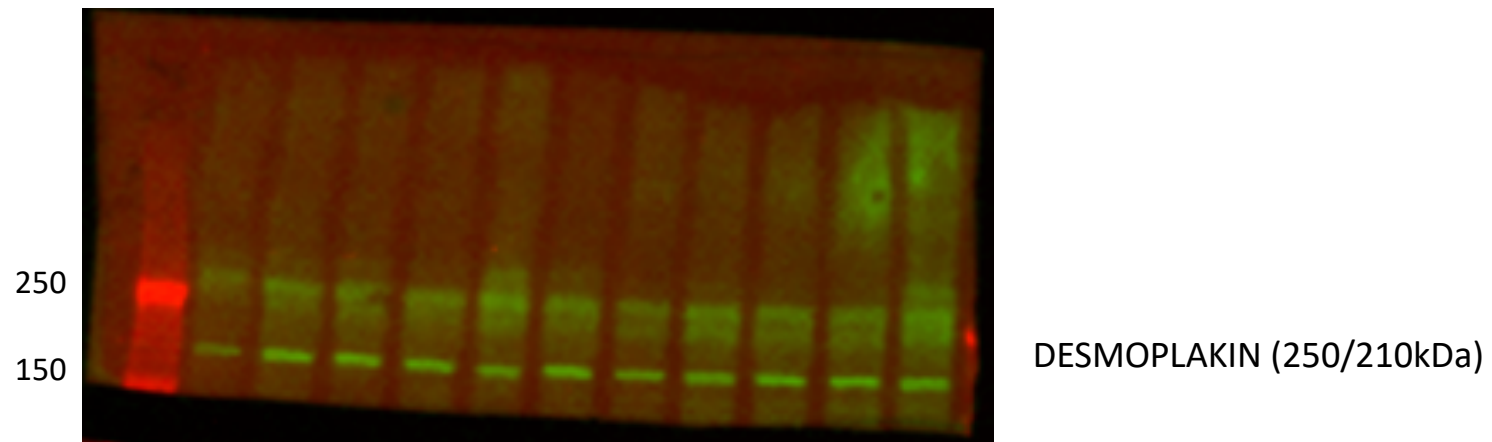

Fig 4D

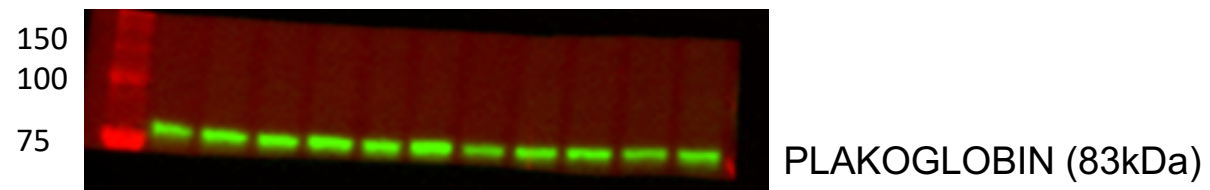

Supplement: Supplementary file 6 — Source Data for Figure 4D [file EMMM-10-e8736-s004.pdf]

Fig 6D

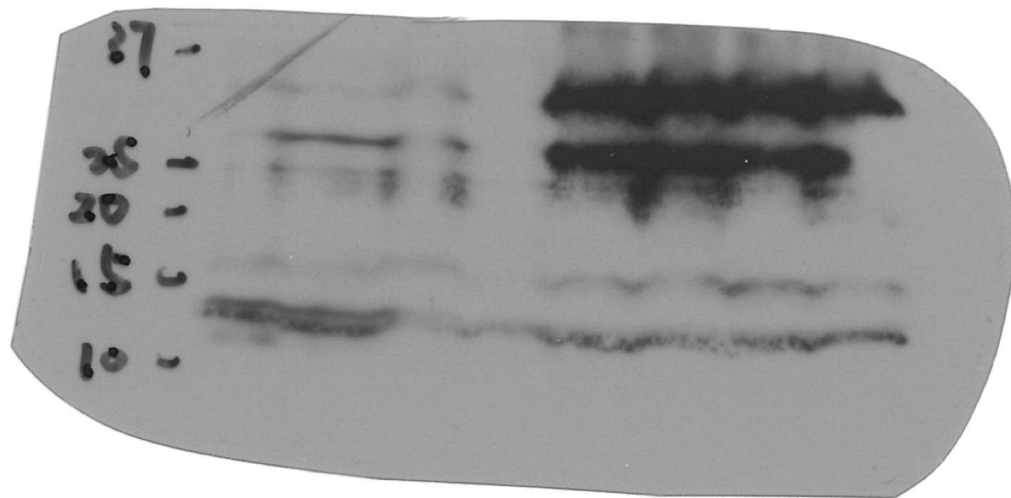

Supplement: Supplementary file 7 — Source Data for Figure 6D [file EMMM-10-e8736-s005.pdf]

Fig 7A

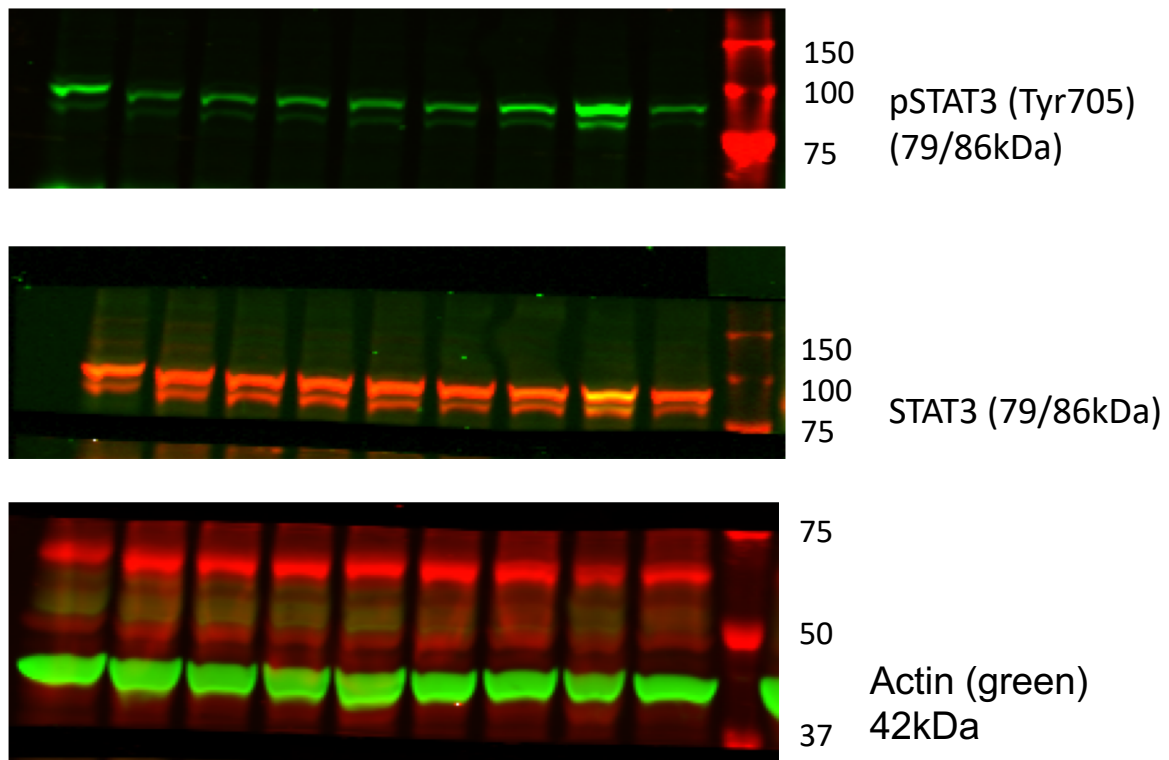

Fig 7A

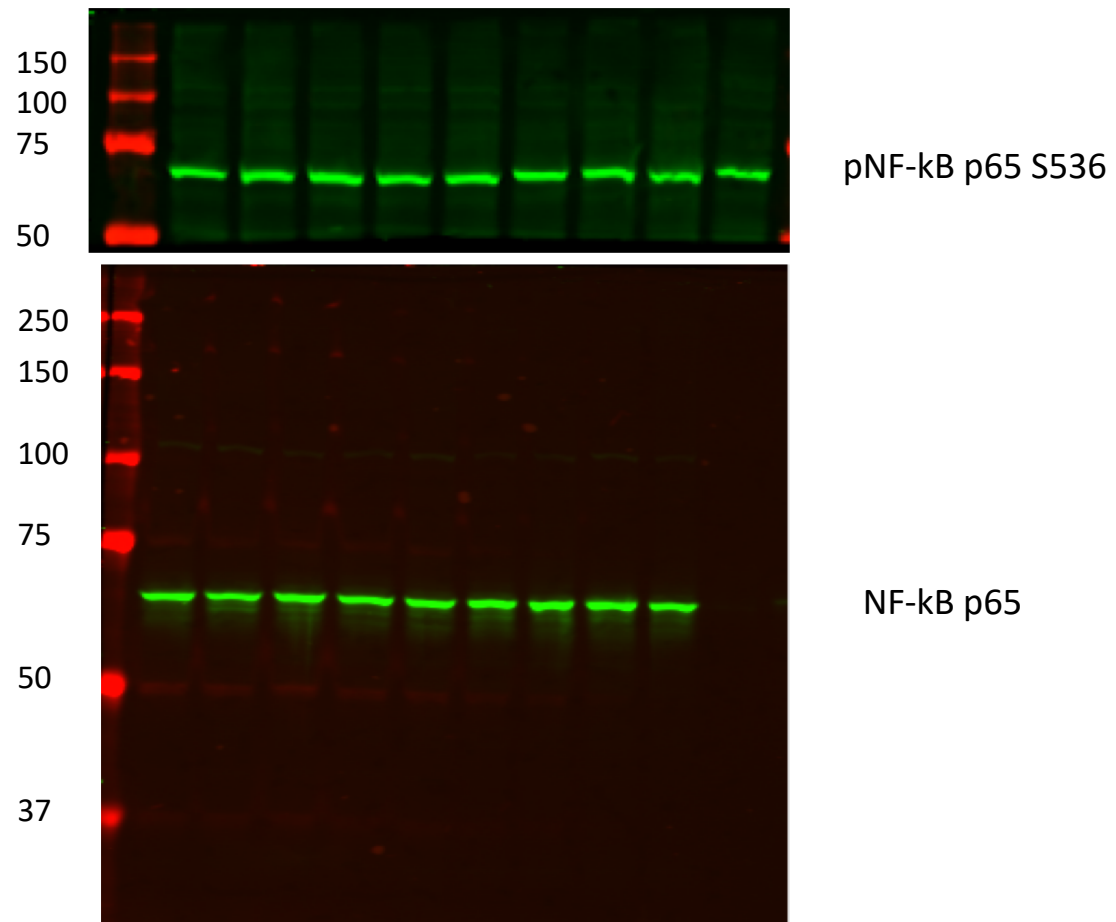

Fig 7F

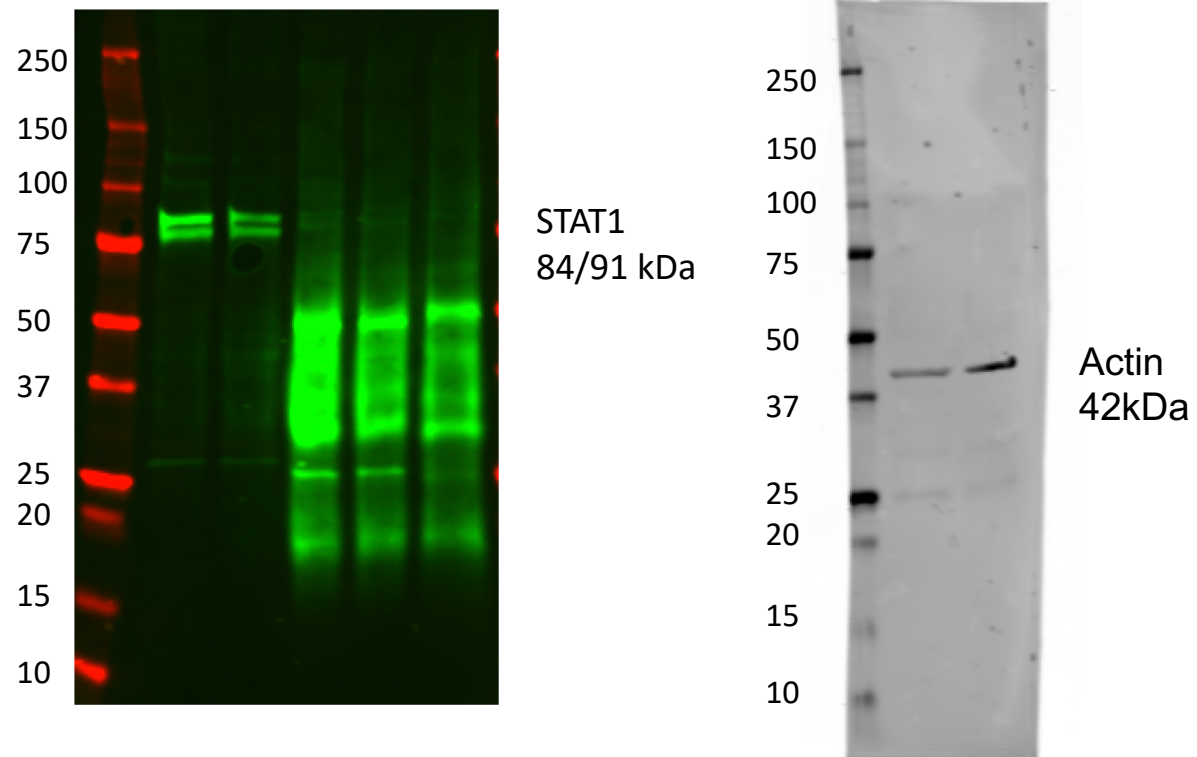

Fig 7G

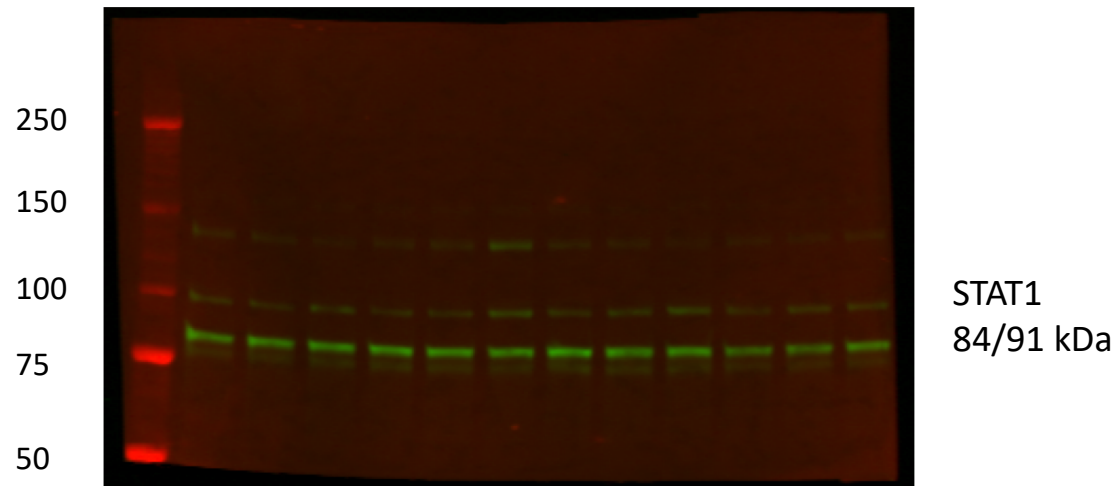

Fig 7G

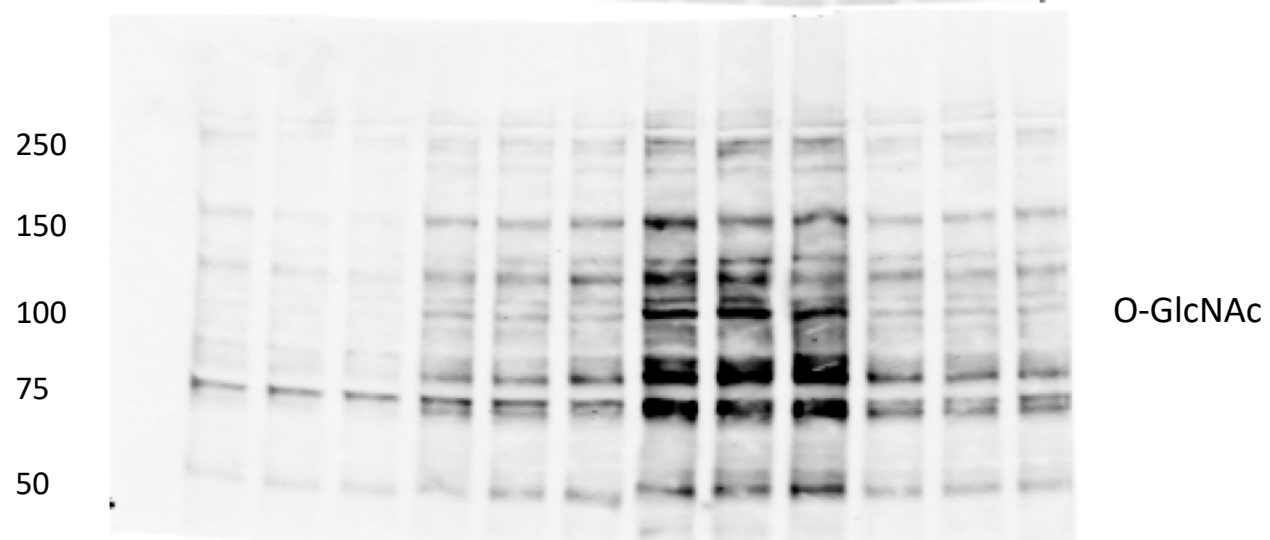

Fig 7G

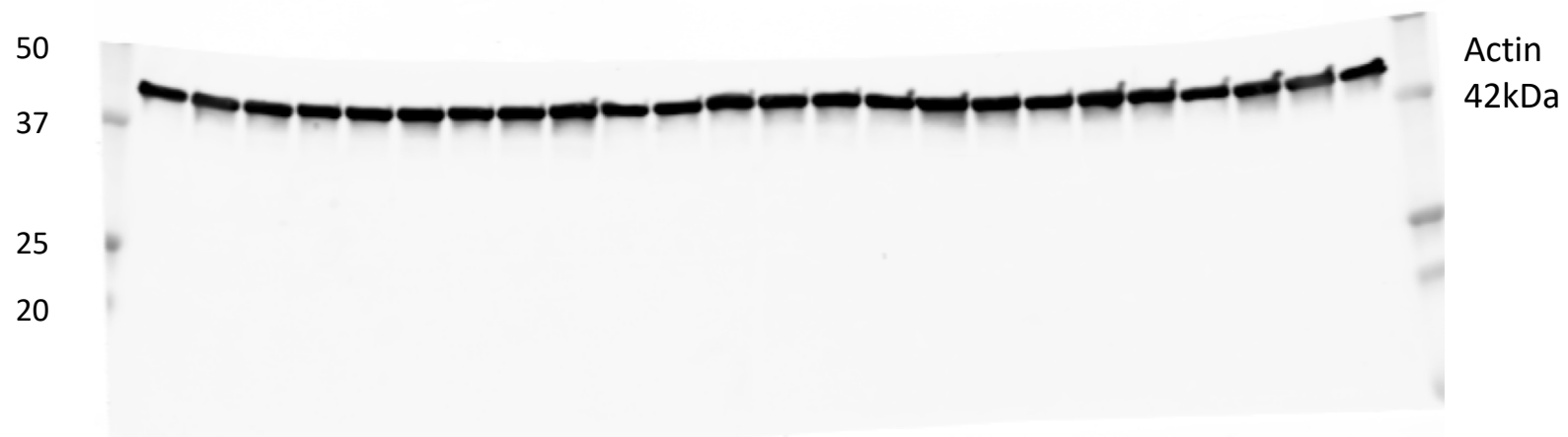

Fig 7H

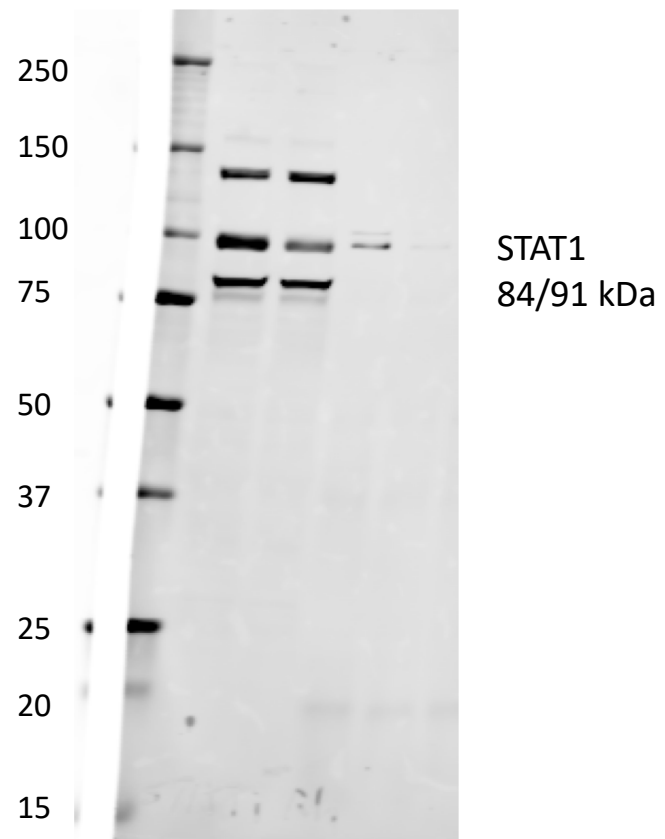

Fig 7I

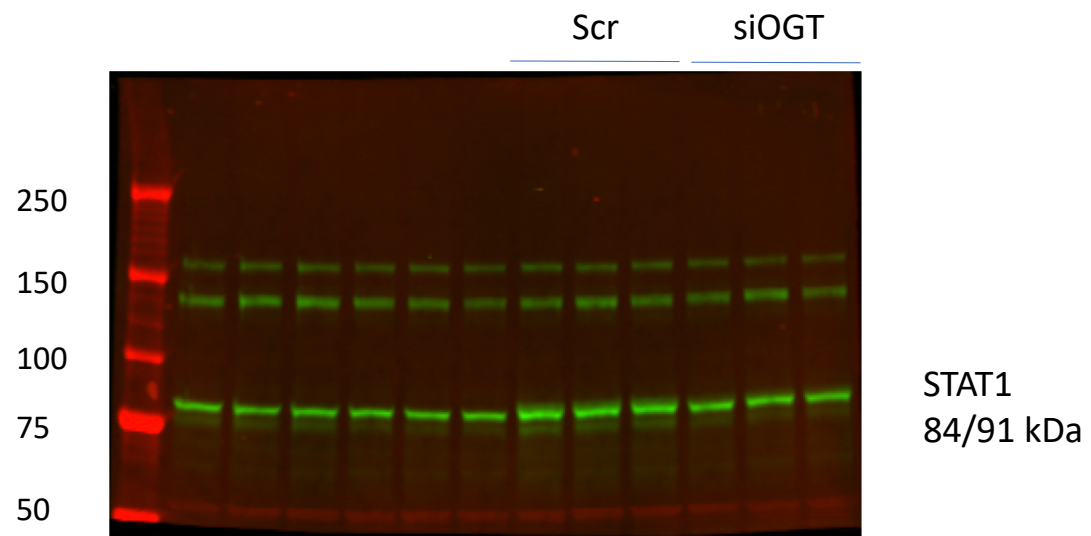

Fig 7I

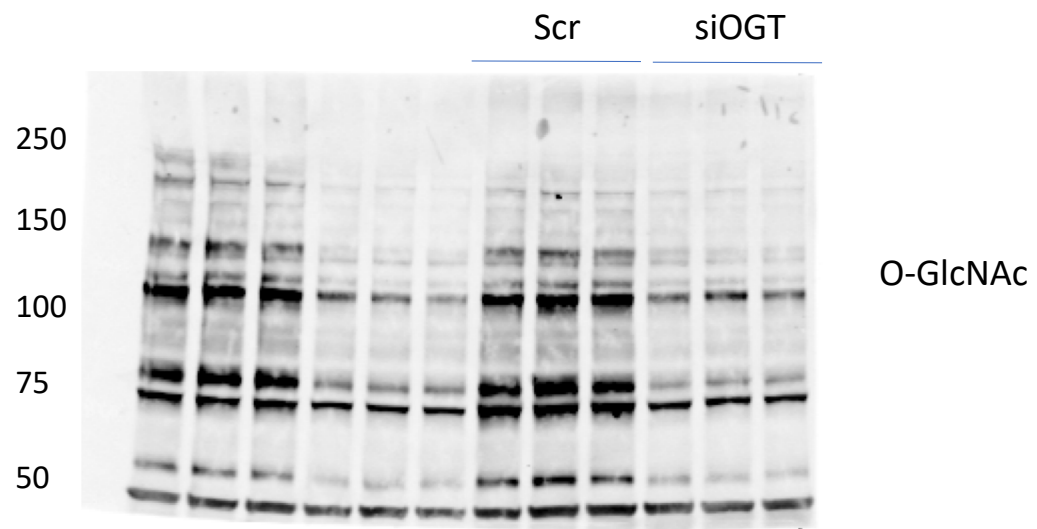

Fig 7I

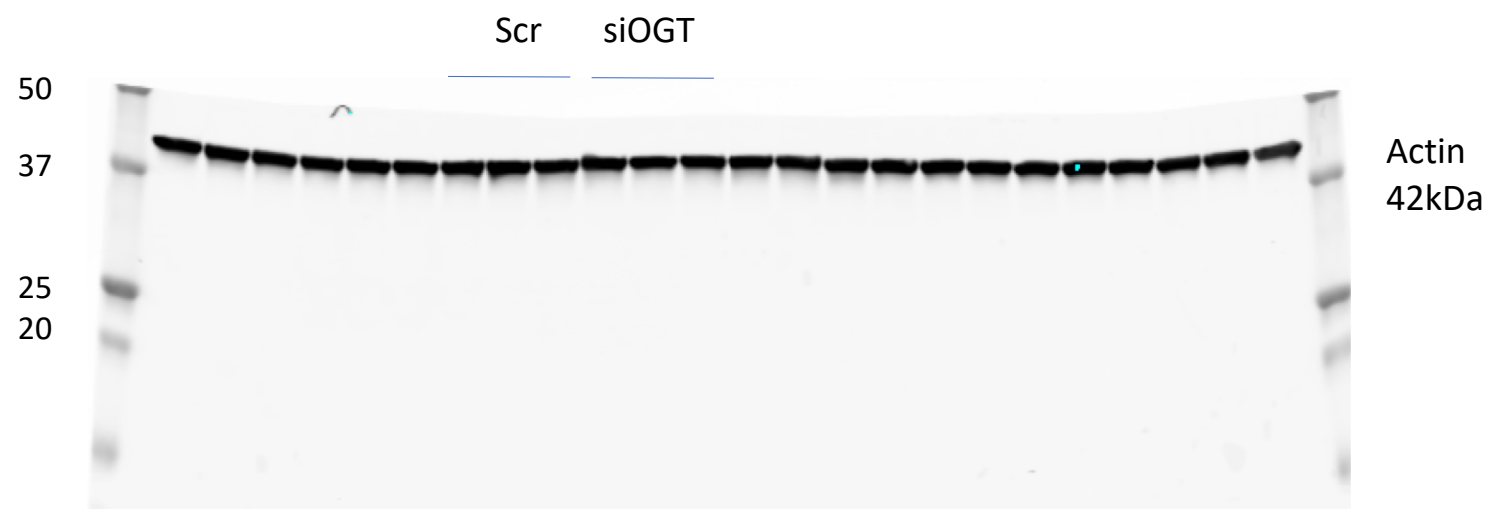

Supplement: Supplementary file 8 — Source Data for Figure 7 [file EMMM-10-e8736-s006.pdf]
